# Structural and immunological impacts of *TOLLIP* nsSNPs: A computational biology approach to drug discovery and immune system modulation

**DOI:** 10.1371/journal.pone.0328573

**Published:** 2025-11-13

**Authors:** Obaid Habib, Saqib Ishaq, Kamran Habib, Aishma Khattak, Wei Yang, Zesong Li, Kainat Bukhari, Amin Ullah, Ajaz Ahmad, Qurban Ali

**Affiliations:** 1 Guangdong Provincial Key Laboratory of System Biology and Synthetics Biology for Urogenital Tumors, School of Basic Medicine, Shenzhen University Medical School, Shenzhen University (SZU), Shenzhen, Guangdong, China; 2 Department of Botany, Khushal Khan Khattak University, Karak (KKKUK), Khyber Pakhtunkhwa, Pakistan; 3 Department of bioinformatics, Shaheed Benazir Bhutto Women University Peshawar, Peshawar, Pakistan; 4 Guangdong Provincial Key Laboratory of Systems Biology and Synthetic Biology for Urogenital Tumors, Shenzhen Key Laboratory of Genitourinary Tumor, Department of Urology, The First Affiliated Hospital of Shenzhen University, Shenzhen Second People’s Hospital (Shenzhen Institute of Translational Medicine), Shenzhen, China; 5 Department of Biotechnology and Genetic Engineering Kohat University of Science and Technology, Kohat Khyber Pakthunkhwa, Pakistan; 6 Department of Allied health sciences, Molecular biology Laboratory, Iqra National University (INU) Peshawar, Peshawar, Pakistan; 7 Department of Clinical Pharmacy, College of Pharmacy, King Saud University, Riyadh, Saudi Arabia; 8 Department of Plant Breeding and Genetics, Faculty of Agriculture Sciences, University of the Punjab, Lahore, Pakistan; Jamia Millia Islamia Central University: Jamia Millia Islamia, INDIA

## Abstract

Toll-Interacting Protein (*TOLLIP*) serves as key adaptor molecule in innate immune signaling, modulating toll-like receptors (TLRs) and interleukin-1 (IL-1) pathway. Despite its central role, the functional impact of non-synonymous single nucleotide polymorphism (nsSNPs) on *TOLLIP* remains unclear. Using an integrated computational approach, we screened 150 *TOLLIP* nsSNPs through consensus predictive tools including PROVEAN, PANTHER, SNPs & GO and SIFT. This approach identified four high confidence deleterious variants (R28Q, T40M, P59L, and R200C) with strong potential to compromise *TOLLIP* protein stability and function. Structural analysis and energy minimization suggested subtle confirmation changes and destabilizing effect, while TM-align displayed preservation of overall folding (TM-score >0.99, RMSD <0.54 Å). Evolutionary conservation, phylogenetic analysis, and protein-protein interaction (PPI) analysis underscored the functional and confirmation importance of these residues. Notably, molecular docking and dynamic simulations revealed that T40M and R200C variants significantly enhance binding affinity for the Afimetoran. Additionally, molecular dynamics (MD) simulations highlighted the altered flexibility, solvent accessibility and modified hydrogen bonds in mutant proteins structure, suggesting potential mechanisms for functional disruption. Collectively, these findings elucidate the structural and functional consequences of nsSNPs on TOLLIP protein stability, and provide a rational base for targeted therapeutic strategies in immune related diseases.

## 1. Introduction

The innate immune system serves as the first line of defense against invasive infection in vertebrates, providing both physical and chemical barriers. Innate and adaptive immunity are collaborating to shield the host from pathogens. Initially, the innate immune system senses and recognizes invariant pathogen associated molecules as foreign entities, whereas the adaptive immune system is highly selective and relies on antigen receptors on the outer surface of B cells and T cells [1]. The human immune system has a collection of specialized recognition transmembrane protein receptors called toll-like receptors (TLRs), which are embedded in the membrane and mainly activated by recognizing structurally conserved motifs by binding to recognition receptors of innate immunity such as damage-associated molecular patterns (DAMPs) and the relatively non-specific, highly conserved pathogen-associated molecular patterns (PAMPs) [2–4]. Upon recognizing the DAMPs and PAMPs, TLRs activate signaling cascades through adaptor proteins, notably the Myeloid differentiation factor 88 *(MyD88*)-dependent-signaling pathway, potentially acts as a down regulator by linking the IL-1 (Interleukin-1) and *TLR* receptor protein to the downstream domain. TLR pathways were first identified in Drosophila and afterward reported in humans [5–7]. Human innate immunity contains ten members of the TLR family, TLR1 to TLR10, each of which is specific and very crucial for a particular kind of immune and cellular response. It induces a different cytokine response [8,9]. TLR6, *TLR*5, TLR4, TLR2, and *TLR*1 are found the plasma membrane of cells, whereas TLR9, TLR8, TLR7, and TLR3 are found on the interior membranes of cells [10–12]. Among the various TLR signaling molecules, Toll-like interacting protein (*TOLLIP*) is a multifunctional immune regulator that down-regulates the TLR signaling pathways. Its primary role is to inhibit excessive pro-inflammatory reactions in innate immunity. The *TOLLIP* also controls the anti-inflammatory factors by suppressing or reducing the pro-inflammatory cytokines such as tumor necrosis factor-alpha (TNF-alpha), interleukin-6 (IL-6), and interleukin-10 [13]. The alteration in the *TLR* pathways genes of a specific host may increase the chance of chronic disease development in the host [14,15]. Genetic diversity refers to the genetic differences within a population through different mutations. It is a crucial parameter in various biological research, especially drug response and disease susceptibility [16]. Variation in the host genetic makeup affects innate immune response and also influences the chances of susceptibility to various diseases. The genetic theory of infectious diseases is a brief history and selected illustrations in human genetics [17,18]. There are many types of variations present in human DNA sequences. Still, the most common are SNPs (single nucleotide polymorphisms), single base pair alterations that account for approximately 90% of human genome variability. Genome-wide prioritization has demonstrated that around 0.12% of the known variations in humans are profoundly harmful [19]. These variations are further classified as non-synonymous SNPs (nsSNPs), non-sense SNPs, coding synonymous SNPs, frameshift variants, non-coding SNPs such as intronic SNPs, 5` UTR (untranslated region), 3` UTR (untranslated region), and splice site SNPs. The nsSNPs, often known as missense SNPs, are thought to play a significant role in altering proteins in the human population because they alter the amino-acid residues by changing DNA base pairs. Which may have a potential neutral or damaging impact on the structure and function of the protein. Disease related and non-lethal nsSNPs can significantly effect on protein structure and function, it may impact the protein in terms of stability, geometry, charge, dynamics, and interactions [20–22]. Consequently, the nsSNPs might have a direct and deleterious effect on the structural and functional integrity of the cell [23]. Several genetic polymorphisms of the *TOLLIP* gene have been reported to be associated as a risk factor in the progression of many diseases, such as cutaneous leishmaniasis [24], visceral leishmaniasis [25], leprosy [26], HIV infection, inflammation [27], tuberculosis [28], malaria [29], sepsis [30], Idiopathic Pulmonary Fibrosis (IPF) [31], and Atopic dermatitis (AD) [32]. Despite known associations between *TOLLIP* polymorphisms and disease susceptibility, the precise impact of nsSNPs on *TOLLIP* structural and functional consequences remains largely uncharacterized. These alterations not only act as biomarker for immune related disorders but also represent a potential target for rational drug targets.

**Fig 1:**
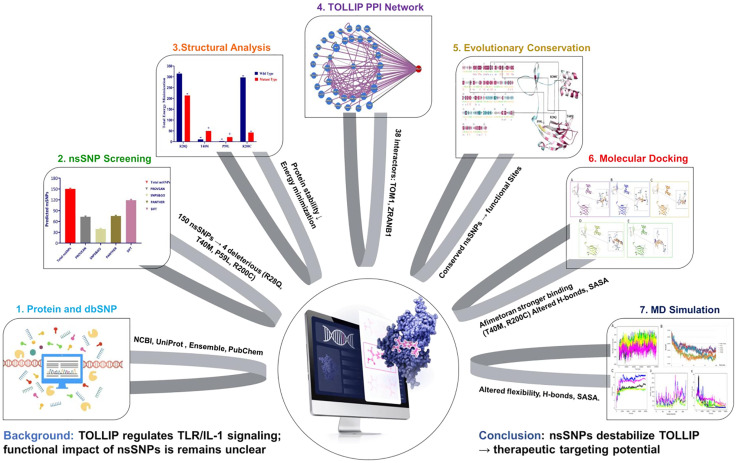
Computational Insights into TOLLIP Protein.

Computational approaches have been widely studied and applied to identify deleterious SNPs in human genes [33], including prediction of deleterious nsSNPs, energy minimization, structural modeling, molecular docking, and dynamic simulations, provide a framework to examine nsSNPs induced perturbations. Moreover, to predict and design ligand capable of modulation mutant protein. Accordingly, we hypothesized that non-synonymous single nucleotide polymorphism (nsSNPs) destabilize TOLLIP protein, impair its regulatory role in *TLR* and IL-I signaling pathways, and we further aimed to systematically explore the target ligand that can rescue mutant *TOLLIP* activity. Thereby, providing insights into both disease mechanism and opportunities for therapeutic targeting. Fig 1 clearly illustrate the key concept of the study, as followed the outlined in [34].

## 2. Materials and methods

**Fig 2:**
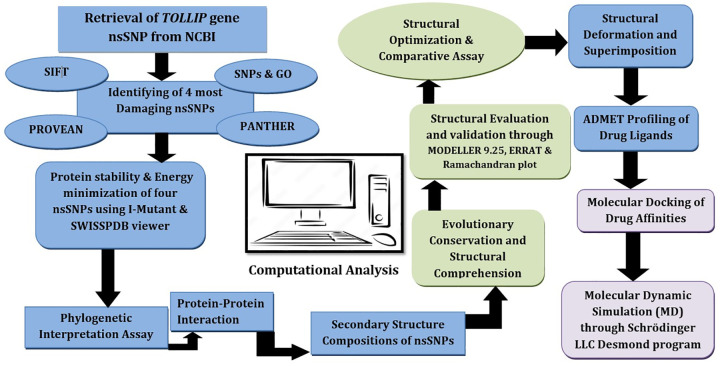
Schematic workflow for analyzing *TOLLIP* nsSNPs and predicting therapeutic ligands.

The detail schematic of the computational analysis performed in this study is shown in Fig 2.

### 2.1. Identification of deleterious non-synonymous single nucleotide polymorphisms

This study assesses the potential effect of non-synonymous SNPs (nsSNPS) on the *TOLLIP* gene confirmation. Initially, we retrieved the FASTA sequence of the TOLLIP protein from the NCBI database (https://www.ncbi.nlm.nih.gov/gene/54472). The accession ID (NP_001305441) and the related missense variants data of the *TOLLIP* gene were obtained and confirmed through the NCBI and UniProt online databases (https://www.uniprot.org/uniprotkb?query=*TOLLIP*). The impact of nsSNPs on the TOLLIP protein was evaluated using various computational tools, included PROVEAN (Protein Variation Effect Analyzer) which assesses the impact of amino acid substitutions on target protein functions [35] (http://provean.jcvi.org/seq_submit.php), PANTHER19.0 (Protein Analysis Through Evolutionary Relationship) [36] which classifies the effects of SNPs based on protein family and function (https://pantherdb.org/), and SNPS&GO is SVM-based classifier which predicts the functional and disease associated variations and its impact on protein stability (https://snps.biofold.org/snps-and-go/snps-and-go.html) [37]. SIFT (Sorting Intolerant From Tolerant) enlisted the genetic variants with predicted effect on protein function, classified as Tolerated and Non Tolerated. The Non Tolerated variants have a high like hood of protein disruptions potential than Tolerated (less likely to be pathogenic) (https://sift.bii.a-star.edu.sg/) [38]. Additionally, we utilized PredictSNP [39] to confirm and verified the selected nsSNPs, which integrates various prediction algorithms to evaluate the effect of nucleotides substitution and pathogenicity of disease-related SNPs (https://loschmidt.chemi.muni.cz/predictsnp/), the predictSNP results are supported by multiple tools, including MAPP, PHD-SNP, Polyphene-1, Polyphene-2, and SNAP.

### 2.2. Protein stability and energy minimization

The detrimental effect of nsSNPs on TOLLIP protein stability was tested on the I-Mutant server (https://folding.biofold.org/cgi-bin/i-mutant2.0.cgi) [40] The I-Mutant server predicts the increase and decrease in protein stability, and the results rely on the RI (reliability index) value, usually set from 0 to 10. The altered protein sequence was capitulated with the default parameters of I-Mutant (Temp = 25 pH = 07). The energy minimization was carried out through computational analysis of the SWISS-PDB server [41] to sort the different energy values of the TOLLIP protein (https://spdbv.unil.ch/). This tool performs computational refinement of protein structures by minimizing the energy to identify the most stable conformation. The energy minimization process involves adjusting atomic positions to reduce the overall energy of the protein structure. This includes molecular properties, such as bond lengths, non-bonded interactions, torsions, electrostatic interactions, angles, and total energy. Energy minimization is a crucial technique in computational modeling.

### 2.3. Phylogenetic analysis of *TOLLIP*

The phylogenetic tree was constructed using the MEGA (Molecular Evolutionary Genetic Analysis) -X tool, which employs Neighbor-Joining (NJ) method to infer the phylogenetic links [42] between the TOLLIP proteins of different species. The Neighbor-Joining method calculates the genetic distance between sequences. It builds a tree based on the shortest cumulative branch lengths, providing insights into the evolutionary history and relatedness of the *TOLLIP* gene across the selected species. The MEGA-X program used Neighbor-Joining techniques to establish the phylogenetic relationship of human *TOLLIP* with 20 other closely related species as identified by the NCBI BLAST program to check the maximum resemblance and confirmation of related gene families in different species.

### 2.4. Protein-protein interaction

Protein-protein interaction (PPI) data of TOLLIP protein was sourced from the Human Reference Protein Interactome Mapping Project (HuRI) via http://www.interactome-atlas.org/. This project systematically interrogates all pairwise combination of human protein-coding genes using high throughput yeast two hybrid screens to identify binary protein interactions [43]. All the interaction *TOLLIP* were extracted, and those with minimum interaction confidence score of 0.6 were selected to ensure high reliability. The Protein-Protein interaction network was constructed using the data sourced from HuRI, allowing for the identification of protein interaction and their association. Additionally, clustering analyses of interaction was analyzed to identify densely connected groups, and key matrices such as node degree and clustering coefficient were calculated to evaluated the networks topology.

### 2.5. Secondary structure characteristics

The SOPMA server was reliable, providing predictions and facilitating subsequent analyses of the *TOLLIP* gene`s secondary structure elements. The *TOLLIP* gene sequence was obtained and submitted to the SOPMA server. SOPMA employs a self-optimized algorithm combining position-specific scoring matrices, multiple sequence alignments, and statistical analysis to predict secondary structure elements. The server-generated predictions for the *TOLLIP* gene`s secondary structure elements, including alpha-helices, beta-strands, and coils. These predictions were compared with experimental data or established methods for validation. The SOPMA results were analyzed to observe the structural characteristics of *TOLLIP* [44].

### 2.6. Evolutionary conservation and structural comprehension of *TOLLIP*

The conservation figuration of *TOLLIP* fragments was examined operating the ConSurf server (https://consurf.tau.ac.il/consurf_index.php), indicating functionally and structurally pivotal corner [45]. The assumption was strengthened through phylogenetic interpretation of firmly associated analogous sequences exploiting a Bayesian inference approach. The ConSurf server designated preservation aggregate assortment from 1 to 9, distributing residues as incredibly conserved 7–9, restrained conserved 5–6, or variable 1–4. This color-coded illustration emphasizes the evolutionary consequence of each residue. Residues in the protein conformity were confidential as either buried within the susceptible or core on the covering. Greatly reclaimed and vulnerable residues are probable functionally notable, whereas preserved and buried residues are additional likely to reinforce to structural durability [46].

### 2.7. Structural evaluation and validation

The **TOLLIP protein** sequence was retrieved and confirmed from the UniProt online database (https://www.uniprot.org/). The BLASTp algorithm was applied to select the basic four templates (1WGL, 1WFJ, 2EP6, and 2NSQ) from the protein databank. Templates were chosen based on sequence similarity to the query sequence, and higher similarity was prioritized. The multi-template structure modeling of the TOLLIP protein was created through the MODELLER 9.25 tool for better and more reliable structure design. The refinement was tested on the CASP 10 method through the Ramachandran plot [47] and ERRAT [48]. The 3D structures and Ramachandran plot were performed to check the phi and psi angles of the protein backbone, ensuring that they fall in a stable and favorable region. For further analysis, the validated 3D structures were refined through RAMPAGE [49] and Galaxy Refine tools [50] ERRAT was used to analyze the overall quality of the target protein structure by analyzing the non-bonding link.

### 2.8. Structural optimization and comparative assay of wild and nsSNPs

The Three-Dimensional (3D) structure sustained energy minimization operating the YASARA (https://www.yasara.org/minimizationserver.htm) software to maximize its structural configuration. This evolution immersed a force field that narrative for hydrogen bonding, electrostatic interactions, van der Waals forces and insuring structural stability and precision. Structural resemblance between the wild and nsSNPs complex was appraised operating TM-Align, which determine a TM-score from (0–1) with scores above (0.5) denoting consequential conformity. The root-mean-square deviation (RMSD) consistently covering from (0.5Å to 4Å), further computed structural distinction with lower values discerning higher similarity.

### 2.9. Structural deformation and superimposition of wild and nsSNPs

The 3-dimensional structures of the wild type and mutant TOLLIP protein were analyzed through Chimera tool [51]. The mutant nsSNPs structures were generated from the wild-type protein by changing the specific altered amino acid at the target position in FASTA sequences. The Chimera interface supports user default commands that are script-based and has the feature of a graphical user interface. The sequence changes were modeled and demonstrated as different rotamers at the target position in *TOLLIP* sequences. Each rotamer revealed the mutation probability in percentage, indicating the likelihood of each rotamer occurring in the target structure. Potential clashes in the structures were identified and resolved. Furthermore, complete and comprehensive mutagenesis analysis and superimposition of the wild-type and mutant structures were performed using Biovia Discovery Studio [52].

### 2.10. Pharmacokinetics evaluation

The pharmacokinetics properties of the four ligands, Afimetoran, Enpatoran, Ruzotolimod, and Ethyl-4-thiadiazole were selected from PubChem (https://pubchem.ncbi.nlm.nih.gov/). The ADMET profile of the Ligand was analyzed through the SwissADME tool. The ChemDraw server was utilized to build structures of the ligands. SMILES (Simplified, Molecular Input Line Entry System) further edited these structures. Good drug candidates possess rapid absorption, even distribution throughout the body, and efficient metabolism to ensure optimal activity [53]. ADMET behavior plays a crucial role in drug development. The impact of toxicity on drug candidates was assessed using CLC-Pred. The pharmacokinetics properties are important because they describe the actual behavior of drugs in the body. The toxicity of drug candidates was evaluated in various cancer types, and the toxicity profile for specific cancer types provided that this step is beneficial for assessing the potential therapeutic drug candidates [54].

### 2.11. Molecular docking (orientation and binding affinity of ligands with target protein)

The Molecular Docking was performed to predict small molecules’ preferred orientation and binding affinity (ligands) with the target protein`s active site. Initially, the three-dimensional (3D) structures of the target protein were generated using different computational tools, such as AlphaFold [55], SWISS-MODEL, Phyre2 [56], and ReptorX. The preparation of the target ligands was carried out for both mutated and wild-type structures through PubChem [57], ChEMBL [58], and Drug Databank. The receptor and ligands were then docked using AutoDock Vina [59], which is commonly used for docking because it’s easy and reliable to use as an interface for visualizing input files and analyzing docking simulations. The docking process ensures the identification of the ligands’ potential binding sites and affinities to the targeted protein receptors.

### 2.12. Molecular dynamic simulation (MD)

The reliable Schrödinger LLC Desmond software was utilized for 100 nanoseconds molecular dynamics simulation [60]. Before MD simulation, TOLLIP protein and ligands docking was carried out to predict the static binding position at the protein active site [61]. MD simulation incorporates Newton’s classical equation of motion, stimulates atomic moment over time, and predicts protein-ligand interaction sites (status) in a physiological environment [62]. The ligand-receptor complex was pre-processed using Maestro’s Protein Preparation Wizard, which included optimization, minimization, and residue completions as needed, with the system built via the System Builder tool. The Intermolecular Interaction Potential 3 Points Transferable (TIP3P) solvent model was utilized in an orthorhombic box with a temperature of 300 K, pressure of 1 atm, and the OPLS_2005 force field [63]. Counter ions and 0.15 M sodium chloride were added to the neutralized models to simulate physiological conditions. The models were kept loosened and relaxed before simulation, and trajectories were stored and used for the inspection every 100 picoseconds.

## 3. Results

### 3.1. Identification of detrimental nsSNPs of TOLLIP protein

Information regarding the TOLLIP protein sequence and 150 non-synonymous SNPs data was retrieved from the NCBI database. The detrimental effect of these nsSNPs on TOLLIP protein structure and function was analyzed via PROVEAN, PANTHER, SNPs&GO and SIFT in silico tools. Out of 150 nsSNPs, the four utilized tools predicted various variants with different frequencies. The 150 nsSNPs were then analyzed on the PROVEAN (Protein Variation Effect Analyzer) tool to determine the deleterious and non-deleterious nsSNPs. The PROVEAN predicted 73 nsSNPs with a deleterious effect. Their final scores were found below the default parameter (−2.5), suggesting they impact TOLLIP protein structure and function. In addition, the PANTHER19.0 (Protein Analysis Through Evolutionary Relationships) tool forecasted 75 disease-associated nsSNPs. The predicted 75 variants’ scores were found to be quite higher, with probably damaging scores than the default score of the PANTHER (0.5) tool. The SNPs&GO server predicted 39 single amino acid polymorphisms (SAPs) likely to be involved in disease progression because the function score of these altered amino acids was calculated greater than the > 0.5 score of SNPs&GO. The SIFT predicted a total of 119 non-tolerated genetic variants and the remaining were found as tolerated. We selected and confirmed the four most common deleterious nsSNPs (rs370240528, rs1180111875, rs772115038, rs780158081) after filtering out on SIFT, PANTHER, SNPS&GO, and PROVEAN. The variant R28Q was predicted to be deleterious with an overall confidence of 61%. This prediction is supported by various multiple tools, MAPP (77%), PhD-SNP (73%), PolyPhen-1 (67%), PolyPhen-2 (47%), SNAP (81%), and SIFT (61%). The T40M variant was predicted with a higher 87% overall confidence, supporting predictions from MAPP (76%), PhD-SNP (59%), PolyPhen-1 (74%), PolyPhen-2 (47%), SNAP (89%), and SIFT (79%). The P59L variant also recorded with 87% confidence, with support of tool predictions MAPP (86%), PhD-SNP (77%), PolyPhen-1 (59%), PolyPhen-2 (56%), SNAP (56%), and SIFT (79%). The R200C mutation was found to be deleterious with a confidence of 61%, supported by MAPP (59%), PhD-SNP (45%), PolyPhen-1 (74%), PolyPhen-2 (47%), SNAP (50%), and SIFT (79%) as described in (Fig S1 in S1 File and Table 1).

**Table 1:** Identification of nsSNPs effect on TOLLIP protein through PROVEAN, PANTHER, SNPS&GO, SIFT.

VARIANT IDS	W/M	PROVEAN	PANTHER	SNPS&GO	SIFT
rs149174945	R28Q	Deleterious −6.001	Probably damaging 0.86	Disease 0.729	Non-Tolerated (1.00)
rs764227120	T40M	Deleterious −7.001	Probably damaging 0.85	Disease 0.778	Non-Tolerated (1.00)
rs1409937148	P59L	Deleterious −6.619	Probably damaging 0.85	Disease 0.746	Non-Tolerated (1.00)
rs144425237	R200C	Deleterious −5.93	Probably damaging 0.86	Disease 0.525	Non-Tolerated (1.00)

### 3.2. Protein stability and energy minimization

The energy minimization and stability of wild-type and mutant TOLLIP proteins were assessed with a clear difference in their resultant values through the I-Mutant server and SWISS-PDB. The I-Mutant2.0 server predicted the change (Increase and decrease) in the stability of mutant protein sequences. The R28Q, P59L, and R200C had decreased stability RI values of 9, 5, and 1, respectively, whereas the T40M variant RI score was 1 with increased stability. The altered protein sequence was capitulated in default parameters of I-Mutant. The energy minimization values for normal and mutant TOLLIP protein were examined, and the total energy value of normal TOLLIP protein was −10744.296 kcal/mol. In comparison, the mutant TOLLIP protein exhibited a total energy of −10855.578 kcal/mol. The clear differences in an average mean value of energy minimization among wild type and mutants (R28Q, T40M, P59L, and R200C), including bonds, angles, torsion, non-bonded, and electrostatic values of residues are shown in (Fig S2 in S1 File and Table 2). The observed differences in stability between the wild-type and mutant TOLLIP proteins significantly highlight the impact of amino acid changes on protein structure, function, and stability.

**Table 2:** Energy minimization comparison between Wild and Mutant (W/M) nsSNPs.

Variants	Bonds		Angles		Torsion		Non-bounded		Electrostatic		Total Value	
W/M	Wild	Variant	Wild	Variant	Wild	Variant	Wild	Variant	Wild	Variant	Wild	Variant
R28Q	1.5	0.98	2.83	2.77	6.23	4.28	−49	−41.7	−277.6	−179.6	−314.3	−213.2
T40M	0.5	1.95	1.94	5.28	0.23	4.6	−16.9	32.8	−24.1	2.97	−10.28	49.57
P59L	1.04	1.83	14.73	10.58	18.75	4.55	−27.3	−14.9	−10.1	18.7	−2.35	21.06
R200C	1.2	0.24	2.93	1.66	4.75	1.71	−34.3	−29.7	−272.3	−16.4	−297.0	−42.01

### 3.3. Phylogenetic analysis of *TOLLIP* gene

Based on BLAST results, the phylogenetic tree was constructed to confirm the conservation *TOLLIP* in other species. Mega-X confirmed high resemblance and evolutionary relationships of 50 known species with human *TOLLIP* gene as illustrated in supplementary (Fig S3 in S1 File). The branch lengths in phylogenetic tree indicated the direct relation to the human *TOLLIP* gene. The results manifest some of the most closely resembled species, including accession IDs XP_050613901.1/XP_050613902.1 (*Macaca thibetana*: Chinese stump-tailed macaque), XP_065383799.1/ EHH55965.1 (*Macaca fascicularis*: Crab-eating macaque), XP_055211765.1 (*Gorilla*: gorilla gorilla/ western gorilla), NP_001248200.1/EHH22555.1 (*Macaca mulatta*: Indochinese rhesus macaque), XP_016775579.1 (*Pan troglodytes*: Chimpanzee), XP_017730175.1 (*Rhinopithecus bieti*: Black-and-white snub-nosed monkey), to the human *TOLLIP* sequence. Some of species were found same with other isoforms of the target protein sequence.

### 3.4. Protein-protein interaction

**Fig 3:**
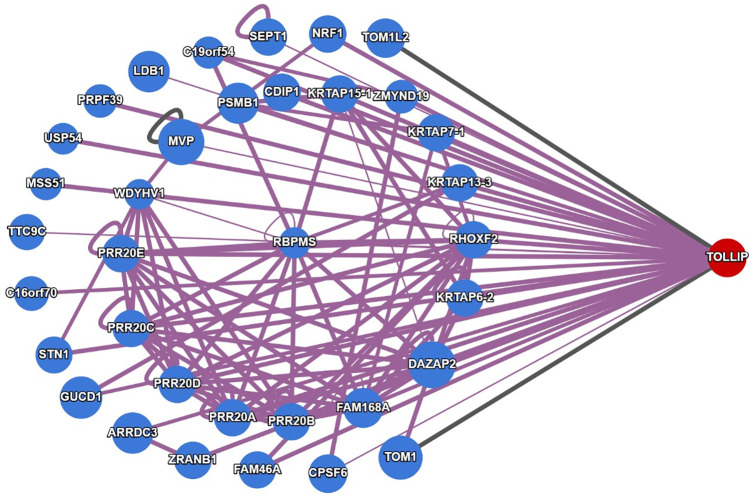
Protein–Protein interaction (PPI) screening of *TOLLIP* illustrating its interrelated proteins and functional association.

The protein-protein (PPI) NETWOEK FOR *TOLLIP* was constructed using data from the Human Reference Protein Interactome Mapping Project (HuRI) and the Interactome Atlas. The analysis revealed 38 direct interactors. Among the key interactors, TOM1, a known interactor [64], exhibited the highest confidence score of 0.917, indicating its potential role in the *TOLLIP* interactome. Other key interactions interactors included PSMB1 (0.671), ZRANB1 (0.906), and CDIP1 (0.823) also revealed highest degree of connectivity within the network. Additionally, NRF1 and MVP, both scoring 0.7, emerged as key proteins in network as depicted in (Fig 3). A pronounced clustering was observed among PRR20 family protein, suggesting a potential collective function. These findings highlight several high confidence interactions of *TOLLIP*, forming a robust network mapping that providing the foundation for further exploration of its functional and molecular associations.

### 3.5. Secondary structure compositions

**Fig 4:**
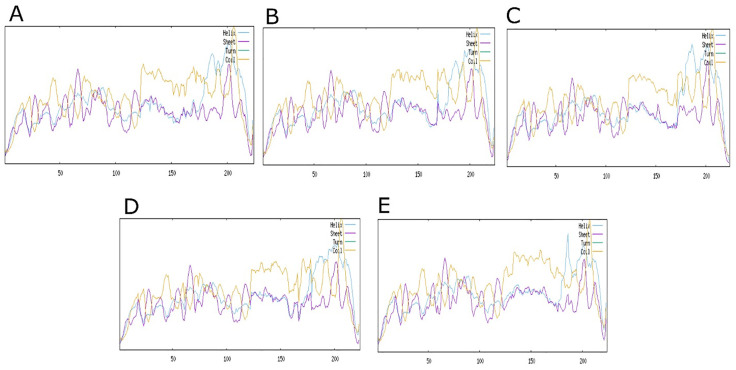
The secondary structural composition of TOLLIP proteins; (A) Wild, (B) R28Q, (C) T40M, (D) P59L, (E) R200C.

The impact of non-synonymous single nucleotide polymorphisms (nsSNPs) on the secondary structure (2D) of *TOLLIP* was scrutinized exploiting the SOPMA server. The wild protein showed a constitution of 24.11% alpha helices, 17.86% extended strands, and 58.04% random coils as illustrated in (Fig 4A). Structural deviation prompted by nsSNPs were appraised, expressive that (Fig 4B) R28Q manifest 21.88% alpha helices, 16.07% extended strands, and 62.05% random coils. (Fig 4C) T40M demonstrated 24.11% alpha helices, 15.62% extended strands, and 60.27% random coils, while (Fig 4D) P59L displayed 21.43% alpha helices, 16.52% extended strands, and 62.05% random coils. In the (Fig 4E) R200C exhibited a more pronounced shift with 20.98% alpha helices, 16.07% extended strands, and 62.95% random coils. Despite inferior divergence in residue composition, the secondary structure (2D) prevailed largely preserved across wild and nsSNPs proteins. The preservation of structural integrity emphasizes the inherent flexibility of the protein and furnished essential justification for further investigations into the functional consequences of these alterations, notably their potential role in disease pathogenesis.

### 3.6. Evolutionary conservation and structural comprehension of *TOLLIP*

**Fig 5:**
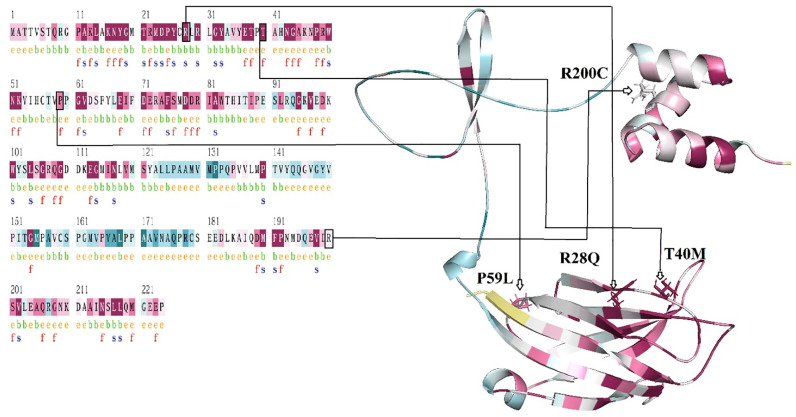
Evolutionary conservation of *TOLLIP* with manipulated nsSNPs (R28Q, T40M, P59L, R200C), highlighting their structural and functional significance.

The evolutionary conservancy of the *TOLLIP* gene was appraised functioning the ConSurf server, which recognizes preserved residues predicated on evolutionary affinity. The four nsSNPs (R28Q, T40M, P59L, R200C) were situated in incredibly conserved areas, detailed their essential in sustaining the protein structural and functional probity. nsSNPs R28Q, T40M, and P59L aggregate a preservation level of (9), while R200C had an outcome of (6), referring inconsiderably inferior conservation (Fig 5). The eminent conservation values for R28Q, T40M, and P59L insinuate these shifts may disintegrate primary protein-protein relation likely remodeling *TOLLIP* function, prohibitively in immune control as illustrated in (Table 3). These examinations emphasize the evolutionary concern to retain these residues, suggesting their crucial features in immune regulation.

**Table 3:** Evolutionary assay of *TOLLIP* nsSNPs, displaying conservation aggregate for each variants and illuminate their consequences.

*rs ID*	nsSNPs	CONSURF SERVER		TM-ALIGN		
		Score	Prediction	TM-Score	RMSD	Prediction
rs149174945	R28Q	9	Strongly conserved and prominently displayed (s)	0.996	0.39	Same fold
rs764227120	T40M	9	Strongly conserved and prominently displayed (f)	0.996	0.43	Same fold
rs1409937148	P59L	9	Strongly conserved and prominently displayed (f)	0.996	0.42	Same fold
rs144425237	R200C	6	conserved and prominently displayed	0.996	0.49	Same fold

### 3.7. Structural evaluation and validation

**Fig 6:**
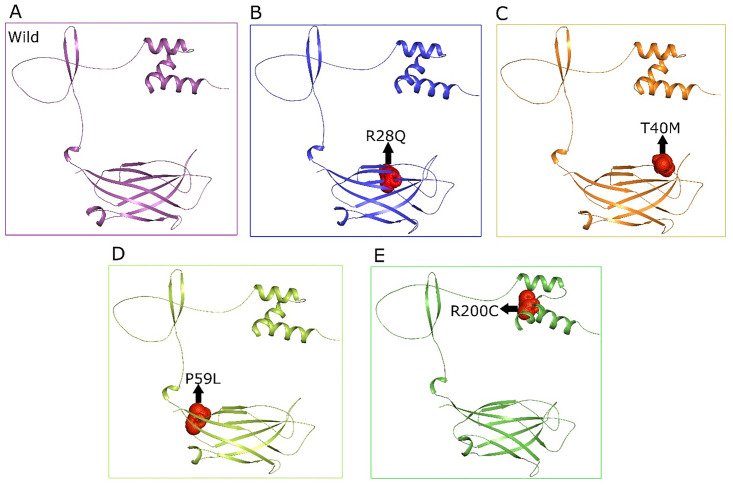
Illustrate the Structural comparisons of Wild and nsSNPs. (A) Wild, (B) R28Q, (C) T40M, (D) P59L, and (E) R200C.

The 3-dimensional structures of TOLLIP Protein were constructed through Modeller9.25 employing numerous templates with the following PDB IDS (1WGL, 1WFJ, 2EP6, and 2NSQ), recruited from the protein databank. The DOPE (Discrete Optimized Protein Energy) default parameters measured the quality of target proteins’ structural models. The DOPE value of −20174.92 was found quite low, which suggested that the protein structure model is likely to be reliable with good quality. Crystallography resolution of the 2EP6 template shows that the targeted protein atomic location range was carried out via X-ray crystallography. Ramachandran plot and EERAT were used for structure validation. Ramachandran plot was examined with 90% of residues in the most favored region, 10% residues in the allowed region, and no fall residues found in the outlier region. The ERRAT provides the quality factor score with a high level of accuracy and reliability of 94.44%. (Fig 6) represents the detailed mutagenesis comparison of both wild and nsSNP (R28Q, T40M, P59L, R200C) structures.

### 3.8. Structural optimization and comparative assay of wild and nsSNPs

Energy minimization for the TOLLIP protein complex was accomplished employing the YASARA to appraise the reliability of wild and nsSNPs (R28Q, T40M, P59L, R200C) entity. Greater integral energy value manifest lower stability in the protein configuration. Before minimization, the whole energy of the wild (*TOLLIP*) complex was −86609.8 kcal/mol, signifying that the original structure apparent some instability. Ensuing energy minimization, the entire energy score of the wild conformity diminish extensively to −113433.3 kcal/mol, exhibiting enhanced durability. Although, the nsSNPs of (R28Q) and (P59L) describing the most notable diminution, extending (−114,422.8 kcal/mol) and (−114,262.3 kcal/mol) respectively, inferring prestigious stabilization as illustrated in (Table 4). The (T40M) and (R200C) alterations showed consequential energy depreciated with conclusive scores of (−112,800.3 kcal/mol) and (−113,200.5 kcal/mol) respectively, deliberating their structural acclimation. These explorations imply that all variants accomplished a staler conformation post-minimization, the confines of stabilization probably domination protein function. Moreover, structural resemblance recruiting the TM-align reveal a higher association between the wild and variants conformity with a TM-score of 0.996 across nsSNPs. (RMSD) aggregate were comparatively moderate with 0.54 Å (R28Q), 0.52 Å (T40M), 0.54 Å (P59L) and 0.51 Å (R200C). These scores intimate minimal structural variance between the wild and variants, verifying that complete protein fold remains exclusive despite the interpolate adaptations.

**Table 4:** Energy Minimization of Wild (*TOLLIP*) and nsSNPs (R28Q, T40M, P59L, R200C) Protein Complex.

*TOLLIP* Gene	TOTAL ENERGY BEFORE MINIMIZATION	ENERGY AFTER MINIMIZATION
^WILD^	−86609.8 kcal/mol	−113433.3 kcal/mol
^**R28Q**^ (rs149174945)	−85938.6 kcal/mol	−114422.8 kcal/mol
^**T40M (**^rs764227120)	−60794.4 kcal/mol	−112800.3 kcal/mol
^**P59L (**^rs1409937148)	−83943.0 kcal/mol	−114262.3 kcal/mol
^**R200C (**^rs144425237)	−86103.7 kcal/mol	−113200.5 kcal/mol

### 3.9. Structural deformation and superimposition of wild and nsSNPS in *TOLLIP*

**Fig 7:**
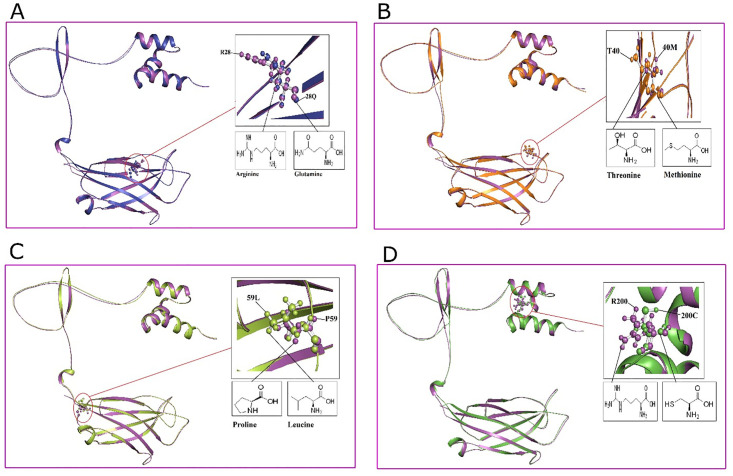
Structural superimposition Wild and nsSNPs of *TOLLIP.* (A) R28Q, (B) T40M, (C) P59L, (D) R200C.

The structural significance of non-synonymous single nucleotide polymorphisms (nsSNPs) in *TOLLIP* were examined through three-dimensional (3D) modeling and interpretation utilizing PyMOL. Non synonymous SNP models were provoke to assess the impact of specific deviations at discrete residue positions, revelatory delicate structural perturbations. Superimposition of wild and nsSNP structures illustrated that the overall protein constitution persisted largely conserved despite these alterations. The root-mean-square deviation (RMSD) scores for the superimposed models were R28Q (0.452 Å), T40M (0.449 Å), P59L (0.451 Å), and R200C (0.435 Å) as depicted in (Fig 7), signifying a high degree of structural accuracy between the variants and the native conformation. While restricted conversions were observed, these mutations lacked generate significant disruption of the protein comprehensive stability, intimating that *TOLLIP* maintains its structural integrity despite sequence variations.

### 3.10. ADMET: Absorption, distribution, metabolism, excretion, and toxicity properties of ligands

The ADMET profiling of the Afimetoran, Enpatoran, Ruzotolimod, and Ethyl-4-thiadiazole revealed the important pharmacokinetic and pharmacodynamic properties. Afimetoran, Ethyl-4-thiadiazole and Enpatoran showed high Gastrointestinal absorption (GI). This indicate that Afimetoran and Ethyl-4-thiadiazole may have good oral bioavailability, whereas Ruzotolimod may require non-oral routes for optimal delivery. Whereas Ruzotolimod exhibited low GI absorption. Afimetoran, Ethyl-4-Thiodiazole, and Ruzotolimod do not permeate the blood-brain barrier (BBB), and minimize the CNS-related side effects, unlike Enpatoran ligand. This idenote a lower risk of CNS-related side effects for the non-BBB-permeable ligands, whereas Enpatoran’s BBB penetration may have central effects that require further evaluation. Afimetoran smaller molecule inhibits CYP2C9, CYP2D6 and CYP2C19. Enpatoran ligand stops the CYP3A4, CYP2C19, and CYP2D6, while the Ethyl-4-Thiodiazole inhibits CYP2C9 and CYP2C19, suggesting strong drug-drug interactions. These outcomes imply possible drug–drug interactions, particularly for Afimetoran and Enpatoran, that should be considered in clinical translation. However, the ligand Ruzotolimod does not affect the CYP enzymes, which clearly indicates interactions of fewer metabolites. The extensive tissue distribution properties were seen in Ethyl-4-Thiodiazole ligand due to the strong (5.33) Log. Overall, Afimetoran and Ethyl-4-thiadiazole emerge as the most promising candidates with favorable ADMET profiles, though metabolic interactions merit further investigation. These investigations suggested that the two drugs Ethyl-4-Thiodiazole and Afimetoran are more significant with potential ADMET properties. The (Table 5) enlisted all the detailed analyses of ADMET properties of selected ligands.

**Table 5:** Evaluation of ADMET profiling of selected ligands on various parameters.

Ligand	Afimetoran	Enpatoran	Ethyl-4-Thiodiazole	Ruzotolimod
**FORMULA**	C26H32N6O	C16H15F3N4	C24H20N2O2S2	C14H18N4O5S
**MW**	444.57	320.31	432.56	354.38
**HEAVY ATOMS**	33	23	30	24
**AROMATIC HEAVY ATOMS**	18	10	18	9
**ROTATABLE BONDS**	5	2	4	5
**H-BOND ACCEPTORS**	4	6	3	7
**H-BOND DONORS**	2	1	0	2
**TPSA**	92.31	65.94	92.5	87.22
**CONSENSUS LOG P**_**O/W**_	3.62	2.53	5.33	0.73
**GI** **ABSORPTION**	High	High	High	Low
**LOG S**	−5.13	−3.69	−6.98	−2.34
**BBB** **PERMEANT**	No	Yes	No	No
**CYP1A2 INHIBITOR**	No	No	No	No
**CYP2C19 INHIBITOR**	Yes	Yes	Yes	No
**CYP2C9 INHIBITOR**	Yes	No	Yes	No
**CYP2D6 INHIBITOR**	Yes	Yes	No	No
**CYP3A4 INHIBITOR**	No	Yes	No	No

### 3.11. Molecular docking

**Fig 8:**
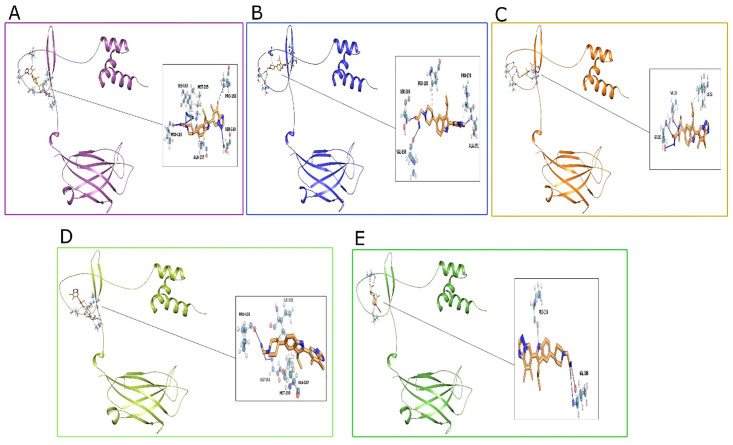
Visualization of best docked molecular interactions of TOLLIP protein with Afimetoran drug. (A) Wild, (B) R28Q, (C) T40M, (D) P59L and (E) R200C.

Molecular docking is a widely used and reliable method for assessing the molecules binding to the target receptors. This study performed molecular docking analysis on the 3-dimensional structure of wild and nsSNPs TOLLIP protein created through Modeller9.25. Out of 44 ligand compounds, the four best candidate drugs were selected based on lower molecular weight: Afimetoran (M.W 444.58), Enpatoran (M.W 320.31), Ruzotolimod (M.W 354.38), Ethyl 4-Thiadiazole (M.W 432.6) as illustrated in (Table S1 in S1 File). Afimetoran and Enpatoran, both (TLR7/8 antagonists in clinical trials) [65,66] alongside Ruzotolimod (TLR7 agonist) [67] enabled antagonist–agonist comparison, while Ethyl 4-thiadiazole served as a structurally distinct comparator. The best binding affinity docking score of all these ligands, with the wild (−6.6 kcal/mol, −5.4 kcal/mol, −5.6 kcal/mol, −5.0 kcal/mol). Furthermore, the docking was operated to determine the binding correlation of Afimetoran drug with wild and nsSNPs variants (R28Q, T40M, P59L, R200C) of the TOLLIP proteins exhibit a lower docking value of −6.6 kcal/mol. Notably, the T40M and R200C SNPs unveiled stronger binding affinities with docking scores of −6.5 kcal/mol and −6.3 kcal/mol respectively. The wild structure exhibited a docking score of (−6.6 kcal/mol) with hydrogen bonds and hydrophobic interactions stabilizing Afimetoran drug in the binding pocket via resides Pro135A, Ile152A, Met155A, Ala157A, Ser160A and Pro165A as depicted in (Fig 8A). The (Fig 8B) R28Q displayed enhanced binding (−6.1 kcal/mol) probable due to strengthened interactions with Val158A, Ser160A, Pro165A, Pro170A and Ala171A. The (Fig 8C) T40M variant, which demonstrated the highest affinity (−6.5 kcal/mol) formed extensive hydrophobic and hydrogen bonding contacts with residues including Ile152A, Val158A and Ser160A, executing a highly stable complex. (Fig 8D) P59L variant showed the affinity (−6.2 kcal/mol) retained key interactions with Pro135A, Ile152A, Gly154A, Met155, Ala157 while the (Fig 8E) R200C (−6.3 kcal/mol) demonstrate notable contacts with Pro156A and Val166A through aromatic stacking contributing to Afimetoran drug stability in the binding pocket. Afimetoran showed the strongest binding to T40M and R200C variants, consistent with its TLR7/8 antagonist profile and suggesting variant-specific modulation of TOLLIP signaling. Ruzotolimod (TLR7 agonist) showed weaker binding, supporting differential agonist–antagonist engagement with *TOLLIP.* The identifying apparent communication are illuminated in the (Table 6) distinguishing insight into the indigenous of Afimetoran drug interaction with wild and nsSNPs proteins complex. The 2D surface around drug ligand entity exhibit (Fig S4 in S1 File) the accurate interaction between the Afimetoran drug and protein-binding site highlighting primary binding residues and interactions. The evaluation offer that distinct SNPs in *TOLLIP*, expressly T40M and R200C enhance Afimetoran drug binding, which may be consequential for targeting these malformations.

**Table 6:** Docking aggregates and elaborate interaction of Afimetoran drug with wild and nsSNPs (R28Q, T40M, P59L, R200C), containing key residues, distances, angles and binding energies.

TOLLIP Protein Complex with Drug (Afimetoran)		Interaction profile			
Protein-Drug complex	Docking score	Residues	Distance (Å)	Donor Angle (°)	Interaction Energy
**WILD**	(−6.6) kcal/mol	Pro135A, Ile152A, Met155A, Ala157A, Ser160A, Pro165A	2.98, 3.52, 3.11, 2.79, 2.89, 3.22	104.36, 174.19, 183.87, 165.55, 111.12, 162.69,	−21.15 (kcal/mol)
**R28Q** (rs149174945)	−6.1 (kcal/mol)	Val158A, Ser160A, Pro165A, Pro170A, Ala171A	2.22, 3.12, 3.11, 2.76, 3.05	161.62, 155.21, 106.12, 152.31, 171.67	−21.14 (kcal/mol)
**T40M (**rs764227120)	−6.5 (kcal/mol)	Ile152A, Val158A, Ser160A	2.88, 3.11, 2.76	145.04, 164.08, 157.47	−24.40 (kcal/mol)
**P59L (**rs1409937148)	−6.2 (kcal/mol)	Pro135A, Ile152A, Gly154A, Met155, Ala157	3.14, 3.52, 2.31, 2.86, 3.11	142.76, 161.65, 157.58, 102.9, 132.21,	−19.70 (kcal/mol)
**R200C (**rs144425237)	−6.3 (kcal/mol)	Pro156A, Val166A	2.44, 1.64	141.33, 152.31	−19.10 (kcal/mol)

### 3.12. MD simulation

#### 3.12.1. Stability analysis (RMSD).

**Fig 9:**
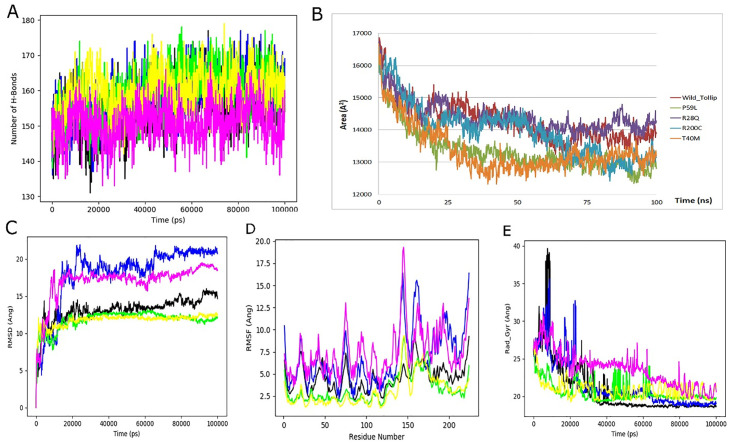
MD simulation analysis of Ca atoms of WT and MT TOLLIP proteins (A) Number of H-bond (B) Solvent-Accessible Surface Area of carbon alpha (C) RMSD plot analysis (D) RMSF per residues (E) Radius of gyration of wild and mutants highlighted through color-coded line graphs for 100 ns.

**Fig 10:**
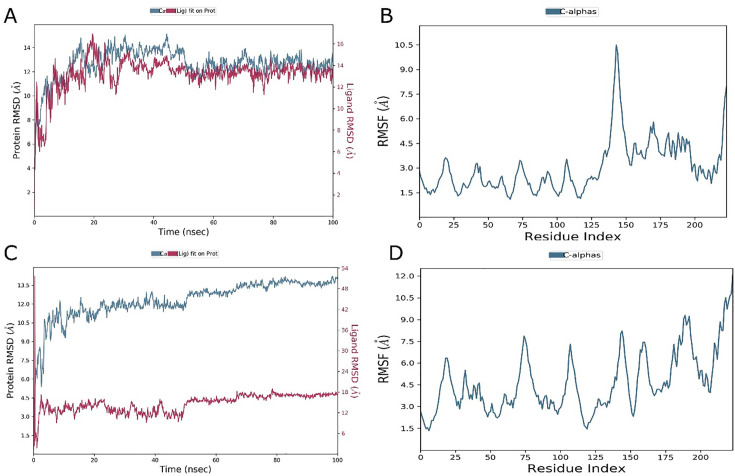
Calculated RMSD values for alpha carbon (Ca) atoms (blue curves) of TOLLIP protein and protein fit Afimetoran ligands (red curves) (A) Wild, (C) Mutant. Line representation of the evolution of Root mean square fluctuation (RMSF) of *TOLLIP* Ca during the MD simulation with Afimetoran drug (B) Wild, (D) Mutant.

The molecular dynamics simulation analysis was conducted to assess the H-Bond, ROG, SASA RMSD, and RMSF. Fig 9C presents the changes in RMSD values over time for the backbone atoms of the non-mutant and mutant proteins. The RMSD value for the predicted proteins fluctuated up to 100 ns during the simulation timeframe, with an average value of 1.2 Å. The RMSD values for the wild type were recorded at 13.14 Å, while in comparison, the RMSD values for mutants were found to be quite higher at residues R28Q (18.08Å), T40M (11.78Å), P59L (11.77Å), R200C (16.99Å), respectively, as demonstrated in Fig 10C. Elevated RMSD value in mutant proteins, suggesting significant structural and functional deviations.

#### 3.12.2. Flexibility analysis (RMSF).

The flexibility analysis of each residue was measured by root mean square fluctuation (RMSF) analysis. The average RMSF value for *TOLLIP* was observed to be 3.79 Å, whereas the average RMSF values for mutant were R28Q (4.91 Å), T40M (2.16 Å), P59L (1.82 Å), R200C (6.29 Å), respectively. All the proteins displayed maintained stability through the 100 ns simulation. However, increased residue flexibility was recorded in mutants. The fluctuation was observed at Glu145 (R200C), Pro224 (R28Q), and Gly148 (T40M and P59L), while the wild type exhibited the highest variation at position Pro224, as shown in Fig 9D.

#### 3.12.3. Hydrogen bond occupancy (H BOND).

Hydrogen bond Occupancy was examined for the assessment and compactness of wild-type and mutant proteins because the hydrogen chemical link plays a vital role in the stability of the amino acid. As shown in Fig 9A, the plot shows the proteins with similar hydrogen bonding. The wild *TOLLIP* averaged 154 H-bonds, while the mutants’ notable average number of hydrogen bonds were 158, 159, and 150, respectively. Change in hydrogen bonding manifests a substantial difference in stability among proteins. The total energy of the system was calculated, to ensure the simulation’s physical validity, no systematic drift was found, and the simulation progress was also compared.

#### 3.12.4. Solvent-accessible surface area (SASA).

The Solvent-Accessible Surface Area, also called aqueous accessibility superficial area analysis, measured the protein around the surface of the aqueous molecules. The SASA mean value of wild-type *TOLLIP* was 14270.42 Å^2,^ where the mutant SASA means values were recorded R28Q (14412.44 Å^2)^, T40M (13365.01 Å^2)^, P59L (13358.10 Å^2)^ and R200C (13989.06 Å^2)^ respectively, (Fig 9B). Throughout the simulations, the notable data was recorded. Initially, during the early stages of the simulation, all the proteins exhibited higher SASA values. However, the values were recorded over time with decreased SASA values, as seen in Fig 9B. The mutant R28Q was notable, with the highest mean SASA value at 14412.44 Å^2^.

#### 3.12.5. Gyration analysis (Rg).

The conformational behavior of the protein was examined by the radius of gyration (Rg) values. Usually, a lower Rg value indicates a more compact structure of protein. The wild *TOLLIP* average Rg value was observed at 20.83 Å, whereas for the mutants, the Rg values were recorded at R28Q (21.46 Å), T40M (20.59 Å), P59L (20.84 Å), and R200C (23.60 Å), respectively. The lowest Rg value was observed in mutant T40M, suggesting the highest degree of compactness among other variants (Fig 9E).

#### 3.12.6. Analysis of protein-ligand interactions and structure stability.

**Fig 11:**
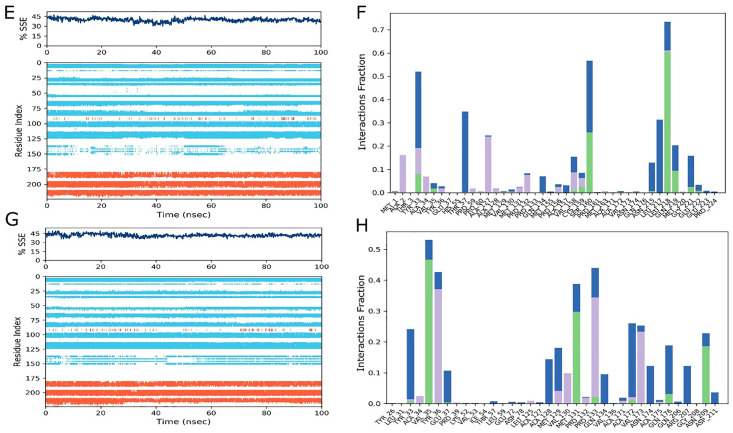
Protein Secondary Structure element (SSE) distribution by residue index throughout the protein structures with Afimetoran drug. Red columns indicate alpha helices and blue columns indicate beta-strands (E) wild and (G) mutant. Ligand-protein binding histogram with Afimetoran drug (F) Wild (H) Mutant.

Figs 10 and 11 illustrates the detailed RMSD and RMSF analysis alongside the secondary structure and interaction with wild and comparative nsSNPs complex structure. The RMSD values in Fig 10A indicate the carbon alpha atoms in ligand-bound *TOLLIP* wild-type protein stabilized at 10 ns, suggesting minimal variation with an RMSD average value of 12.66 Å within a range of 3.0 Å. The selected drug stability was also achieved after 40 ns, with constant RMSD values with the final 100 ns, exhibiting equilibrium balance. However, Minor RMSD structural variations indicate potential and possible binding changes in state. Fig 10C revealed and indicated the mutant structure with the first ligand for the *TOLLIP* gene; the values of RMSD were found stabilized at 20 ns with a structural variation of 12.29 Å RMSD value within 1.0 Å (over 80 ns). There was a little increase in RMSD values at the end of the mentioned period, with minimal fluctuations suggesting stability. The ligand recorded the equilibrium within 10 ns, indicating maintained RMSD. Fig 10B and 10D showed RMSF values of 2.94 Å and 4.41 Å, respectively, highlighting the change interactions of ligands with different protein structures. The decreased RMSF at binding sites reflects the stable attachment of ligands, while the higher peaks in loop regions and terminal zones highlight the final stability. Fig 11E and 11G show the confirmed Secondary structure composition (3.67% alpha helices, 46.21% beta sheets, and 4.2% other elements, totaling 54.08% secondary structure. Fig 11F and 11H illustrated that hydrogen and hydrophobic binding were necessary for ligand-protein interaction, with detailed interactions between ligand atoms and protein residues. Importantly, the Ligand Interaction Diagram (Fig 11F, 11H) highlights persistent hydrogen bonds, hydrophobic contacts, and water bridges between Afimetoran and key TOLLIP residues throughout the 100 ns simulation, confirming stable ligand binding and supporting the docking predictions.

## 4. Discussion

Toll interacting protein (*TOLLIP*) is an important negative regulator protein in *TLR* signaling cascade, playing a key role in modulating innate immune responses, including IL-1 mediating pathways. The *TOLLIP* gene is located on chromosome 11p15.5 and consist of 11 exons, spliced to multiple transcript isforms that encode different TOLLIP protein variants. Dysregulations of *TOLLIP* gene, particularly genetic polymorphisms have been reported in susceptibility to and progression of distinct chronic infectious and fibrotic diseases, including Tuberculosis [68], malaria [69], Leprosy disease [70,71], visceral leishmaniasis [72], cutaneous (cuticle layer) leishmaniasis [24], and idiopathic pulmonary fibrosis lung disease (IPF) [73]. Despite these association of variants, the precise molecular mechanisms through specific nsSNPs affects *TOLLIP* function remain poorly understood. The current study addressed this gap by integrated comprehensive computational approaches, including in silico-based prediction, conservation analysis, energy minimization, and molecular modelling, and molecular dynamics. This allowed us to identifying most deleterious nsSNPs in the human TOLLIP protein, that effect its structure and function. Total of 150 nsSNPs of TOLLIP protein were screened via predictive and structural tools, leading to the identification of four confirmed high confidence deleterious variants: R28Q (rs149174945, T40M (rs764227120), P59L (rs1409937148), and R200C (rs144425237). These variants are predicted to compromise TOLLIP protein Stability and may potentially impair its regulatory function. Multiple prediction tools confirmed their deleterious potential, with overall confidence scores ranging from 61% to 87%. The three variants R28Q, T40M, and P59L were predominantly located in highly conserved region (ConSurf Score 09), indicating their functional importance and protein structure destabilization. The findings were found align with previous report displaying that nsSNPs in conserved regions of immune regulatory proteins, such as MyD88 and IRAK4 disrupt protein stability and function, increasing susceptibility to infectious disease [74,75].

The current study builds on these comprehensive workflows, to examine how specifically nsSNPs can impact protein, particularly structural changes that influence targeted protein dynamics and ligand interaction. Integrating sequences and structural context computational predictions [76]. These parallels approaches highlighted the validity of our workflow in identifying functionally relevant to non-synonymous SNPs roles. Furthermore, MD simulations supported these predictions, showing increased flexibility, alteration in RMSD/RMSF values, and a clear shift were observed in solvent accessible surface area relative to wild type *TOLLIP*. These structural destabilization via nsSNPs are observed and reminiscent in adaptor and signalling proteins *IRAK1* and *TRAF6*, where nsSNPs altered structure and disrupt function and downstream signalling, underscoring the potential for similar mechanistic consequences in *TOLLIP* [77,78]. The results were align with, a comprehensive computational study that investigated 19 deleterious nsSNPs in the *CTNS* gene, that exhibited alteration in cystinosin stability and function, with molecular dynamics confirming structural disruption of conserved Sites [79]. Similarly, studies was reported on IL1B1 gene, using multiple predictive and structural tools, along with molecular dynamic simulation have confirmed that nsSNPs can potentially induce instability and alter flexibility [80]. Furthermore, previous computational studies on proteins, such as cyclin dependent kinase 2 (*CDK-2*) and *HsaA* monooxygenase 2 have employed molecular docking ADMET profiling, and molecular dynamic simulation to assess the impact of variants or as inhibitor binding, with a primary focused on investigation of potent and significant ligands and evaluating the selected ligands stability [81–84].

**Fig 12:**
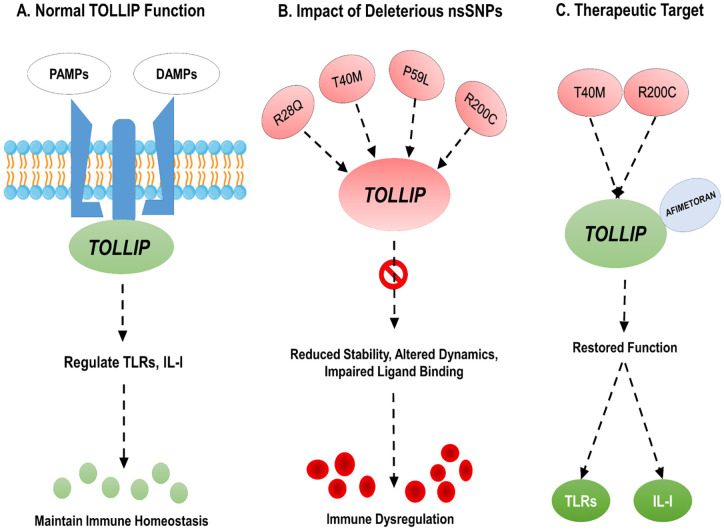
Schematic representation of TOLLIP function, impact of deleterious nsSNPs and potential therapeutic strategy.

Both in silco and experimental studies across multiple proteins examinations revealed that missense substitutions often alter protein stability, leading to loss of function and impaired downstream signalling, further supports our findings [85,86]. Importantly, the molecular docking study further confirmed that afimetoran binds strongly to both mutant and wild type TOLLIP protein, and afimetoran has been recently reported in clinical study of Cutaneous Lupus Erythematosus, as toll like receptor 7/8 (*TLR*7/8) antagonist [87]. Moreover, observed docking scores of Afimetoran (−6.6 kcal/mol), Enpatoran (−5.4 kcal/mol), and Ruzotolimod (−5.6 kcal/mol) are consistent with their known pharmacological profiles. Afimetoran, a potent TLR7/8 dual antagonist, exhibited higher binding affinity, which is align with its in vitro efficacy in inhibiting cytokine production through TLR7/8 [88]. Enpatoran with same similar TLR7/8 antagonist profile showed moderate binding affinity, correlating its potential in autoimmune disorder. Enpatoran, also a TLR7/8 antagonist, exhibited moderate binding affinity, which correlates with its therapeutic potential in autoimmune diseases as reported in EMD Serono. (2025, May 21). Ruzotolimod (TLR7 agonist), demonstrated weak binding, aligning with its profile as an oral immunomodulatory activating TLR7 [67].The afimetoran, alone or in combination with prednisolone, showed efficacy in NZB/W mice lupus model, and successfully reduced sensitivity of bone marrow pDC and B cells to prednisolone induce apoptosis [89], which further support our findings. Similar outcomes have been observed in *TLR*9 and NOD2, where nsSNPs regulated ligand binding interaction, suggesting potential personalized therapeutic strategy. The ADMET profiling of afimetoran and ethyl 4 thiadiazole possess favourable absorption, distribution and low toxicity. The ADMET analysis provide valuable insight into pharmacokinetics and emphasize that nsSNPs could induced changes in ligand binding, ultimately alter the drug’s efficacy or metabolism. Beyond ligand protein interaction, *TOLLIP* network topology and clustering indicates that it plays a crucial role in cellular homeostasis and co-regulatory functions. The identification of multiple high-confidence interactors for *TOLLIP* highlights its potential role in diverse cellular pathways and regulatory mechanisms. Notably TOM1, a key interactor in PPI network, may influence or regulated immune associated responses, which may possibly be influencing trafficking and immune responses [64]. Structural destabilization of *TOLLIP* nsSNPs may not only weaken its own dogma, but also interfere protein–protein network, that are crucial for immune responses. Similar effects have been observed in *TRAF6* and *IRAK1* (adaptor proteins), where nsSNP-induced structural alterations impair their interaction networks, ultimately disrupting downstream *TLR* signaling [90–94]. Although this comprehensive computational study provides a mechanistic and valuable insights into the impact of deleterious nsSNPs on TOLLIP protein stability and potential ligand interaction. But it cannot fully reflect the complexity of cellular and organism contexts. Moreover, the functional impact of identified deleterious nsSNPs on TOLLIP protein, as well as the efficacy and safety of candidate drugs, remain experimental validation in both in vitro and in vivo models to confirm their translational relevance. Fig 12, illustrate the TOLLIP function, impact of deleterious nsSNPs and potential therapeutic strategy.

## 5. Conclusion

Our integrative computational investigation demonstrates that four deleterious non-synonymous SNPs (R28Q, T40M, P59L, R200C) prompt noticeable structural and functional changes in the TOLLIP protein, compromising its stability and interactions within immune response pathways. The R28Q, T40M and P59L nsSNPs shifts were particularly destabilizing, effecting protein dynamics and protein ligand interactions. Molecular docking and dynamic simulations (MD) further revealed altered ligand binding and solvent accessibility in the variant proteins. Consistent with previous studies [95] showing that even minor sequence changes, such as a single base pair shift, can dramatically alter protein interactions, our results highlight the variant-specific modulation of TOLLIP function. Overall, these findings suggest that genetic substitutions could influence immune regulations and provide insight for potential therapeutic treatment.

## Supporting information

S1 File Supplementary File of TOLLIP. (DOCX)

## Abbreviations

TOLLIP: Toll-Interacting Protein
TLR: Toll-Like Receptor
PAMPs: Pathogen-Associated Molecular Patterns
DAMPs: Damage-Associated Molecular Patterns
IL-1: Interleukin-1
IL-6: Interleukin-6
nsSNP: Non-Synonymous Single Nucleotide Polymorphism
SNP: Single Nucleotide Polymorphism
MD: Molecular Dynamics
RMSD: Root Mean Square Deviation
RMSF: Root Mean Square Fluctuation
SASA: Solvent Accessible Surface Area
GO: Gene Ontology
3D: Three-Dimensional
KEGG: Kyoto Encyclopedia of Genes and Genomes
STRING: Search Tool for the Retrieval of Interacting Genes/Proteins
PROVEAN: Protein Variation Effect Analyzer
SIFT: Sorting Intolerant From Tolerant
PolyPhen-2: Polymorphism Phenotyping v2
CADD: Combined Annotation Dependent Depletion
dbSNP: Single Nucleotide Polymorphism Database
ADMET: Absorption, Distribution, Metabolism, Excretion, Toxicity
PPI network: Protein–Protein Interaction network
BLAST: Basic Local Alignment Search Tool
FASTA: Fast-All
HIV: Human Immunodeficiency Virus
IPF: Idiopathic Pulmonary Fibrosis
MyD88: Myeloid Differentiation Primary Response 88
RAK1: Interleukin-1 Receptor-Associated Kinase 1
IRAK4: Interleukin-1 Receptor-Associated Kinase 4
TRAF6: TNF Receptor-Associated Factor 6
TOM1: Target of Myb1
NF-κB: Nuclear Factor kappa-light-chain-enhancer of activated B cells
STRING: Search Tool for the Retrieval of Interacting Genes/Proteins
PROVEAN: Protein Variation Effect Analyzer
SIFT: Sorting Intolerant From Tolerant
PolyPhen-2: Polymorphism Phenotyping v2
CADD: Combined Annotation Dependent Depletion
dbSNP: Single Nucleotide Polymorphism Database
ClinVar: Clinical Variations Database
