# Supplementary material for: Structural and immunological impacts of TOLLIP nsSNPs: A computational biology approach to drug discovery and immune system modulation
Source: PLoS One. 2025 Nov 13;20(11):e0328573. doi: 10.1371/journal.pone.0328573 (PMC12614547; doi:10.1371/journal.pone.0328573)
Supplement: S1 File — (DOCX) [file pone.0328573.s001.docx]

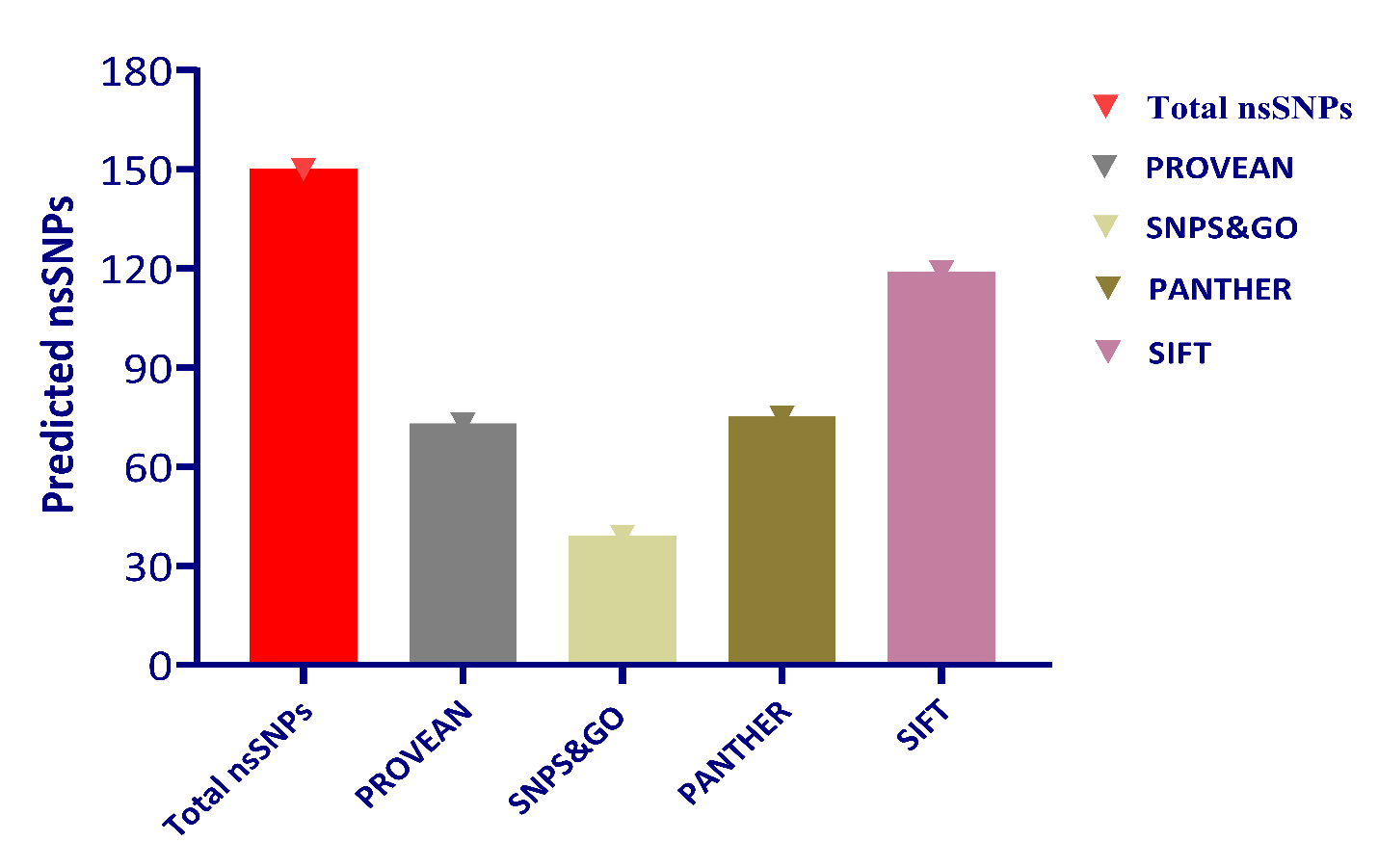
**Figure S1:** The predicted results of PROVEAN, PANTHER, SNPS&GO, SIFT.


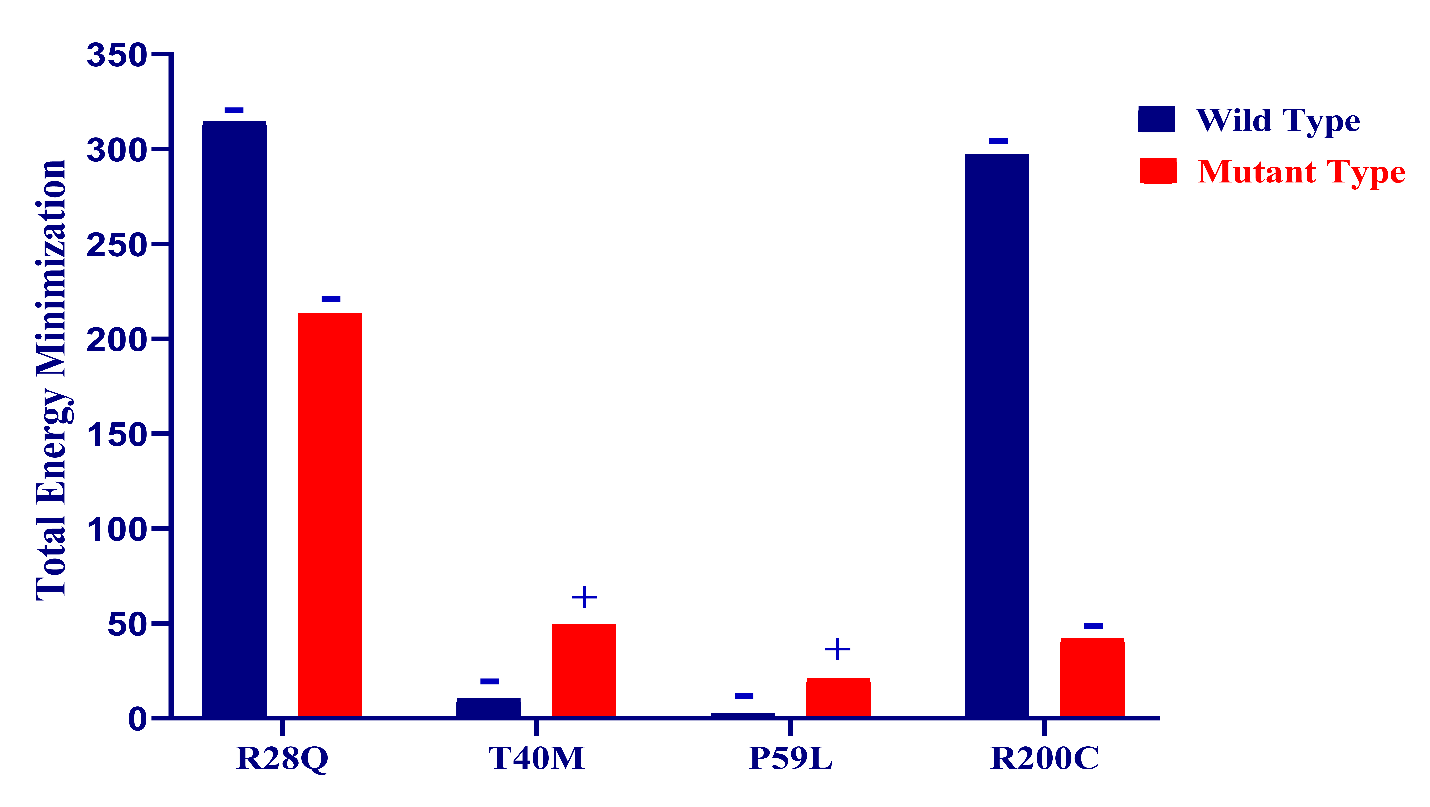


**Figure S2:** Energy minimization of wild and nsSNPs TOLLIP protein, illustrating stability refinement after minimization.


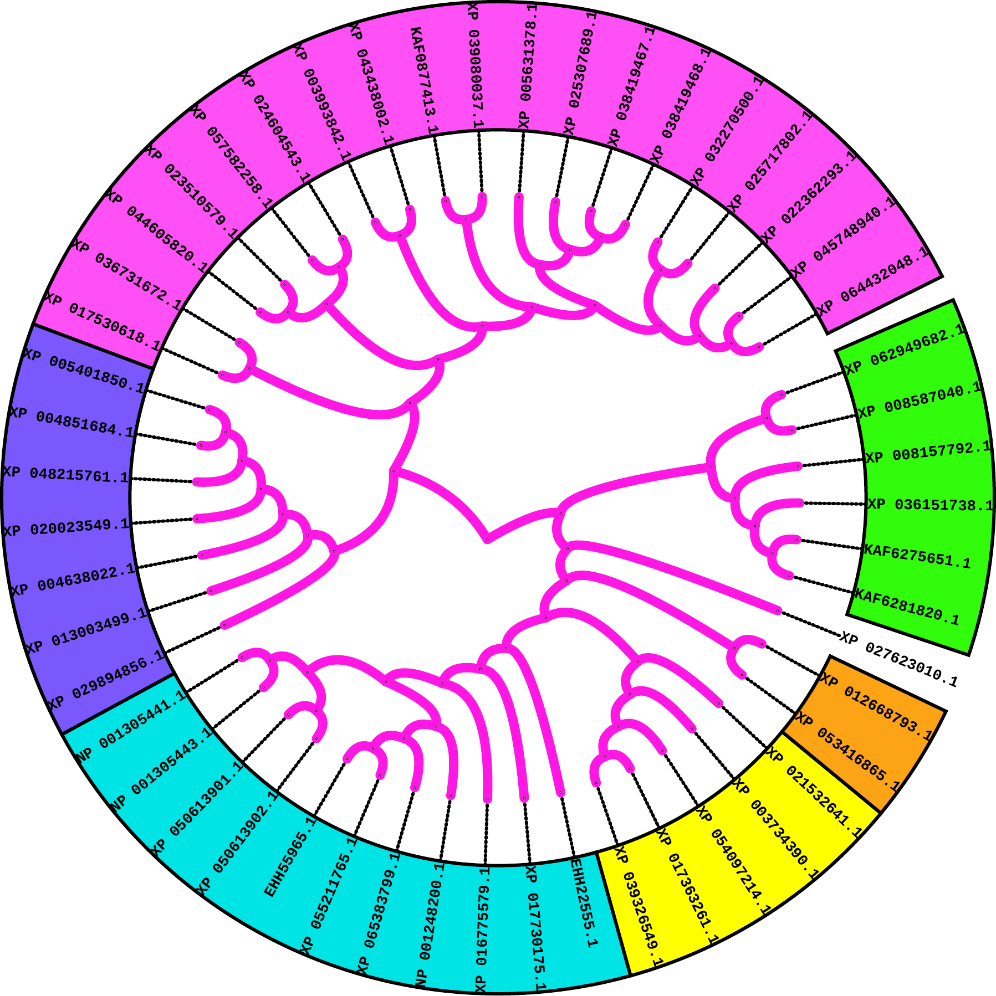


**Figure S3:** Phylogenetic tree of *TOLLIP*.

**Table S1:** Comprehensive the 4 Conclude Screening of Drug Candidates Targeting for *TOLLIP*

| **Serial NO** | **DRUG LIGANDS** | **2D**  **STRUCTURES** | **3D**  **CONFORMER** | **SMILES** | **MOLECULAR WEIGHT** | **MOLECULAR**  **FORMULA** |
| --- | --- | --- | --- | --- | --- | --- |
| **1** | Afimetoran | 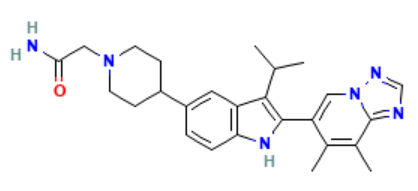 | 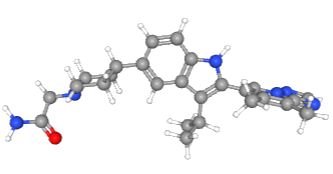 | CC1=C(C2=NC=NN2C=C1C3=C(C4=C(N3)C=CC(=C4)C5CCN(CC5)CC(=O)N)C(C)C)C | 444.58 g/mol | C26H32N6O |
| **2** | Enpatoran | 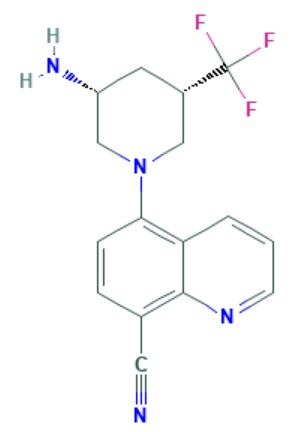 | 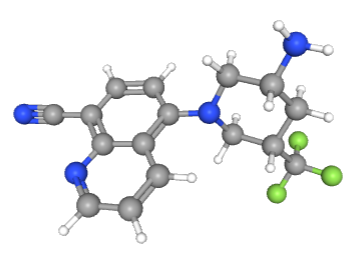 | C1[C@@H](CN(C[C@@H]1N)C2=C3C=CC=NC3=C(C=C2)C#N)C(F)(F)F | 320.31 g/mol | C16H15F3N4 |
| **3** | Ruzotolimod | 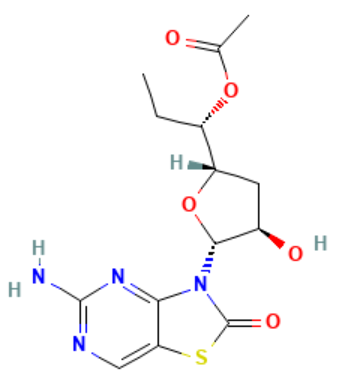 | 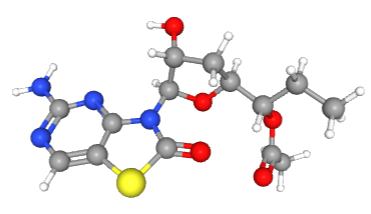 | CC[C@@H]([C@@H]1C[C@H]([C@@H](O1)N2C3=NC(=NC=C3SC2=O)N)O)OC(=O)C | 354.38 g/mol | C14H18N4O5S |
| **4** | Ethyl 4-Thiadiazole | 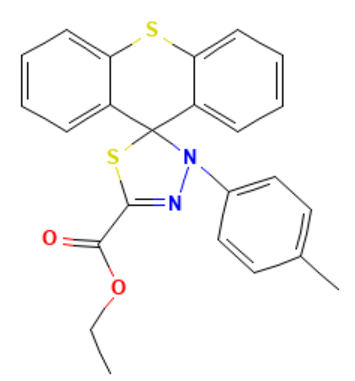 | 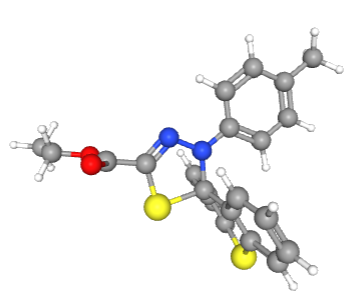 | CCOC(=O)C1=NN(C2(S1)C3=CC=CC=C3SC4=CC=CC=C24)C5=CC=C(C=C5)C | 432.6 g/mol | C24H20N2O2S2 |


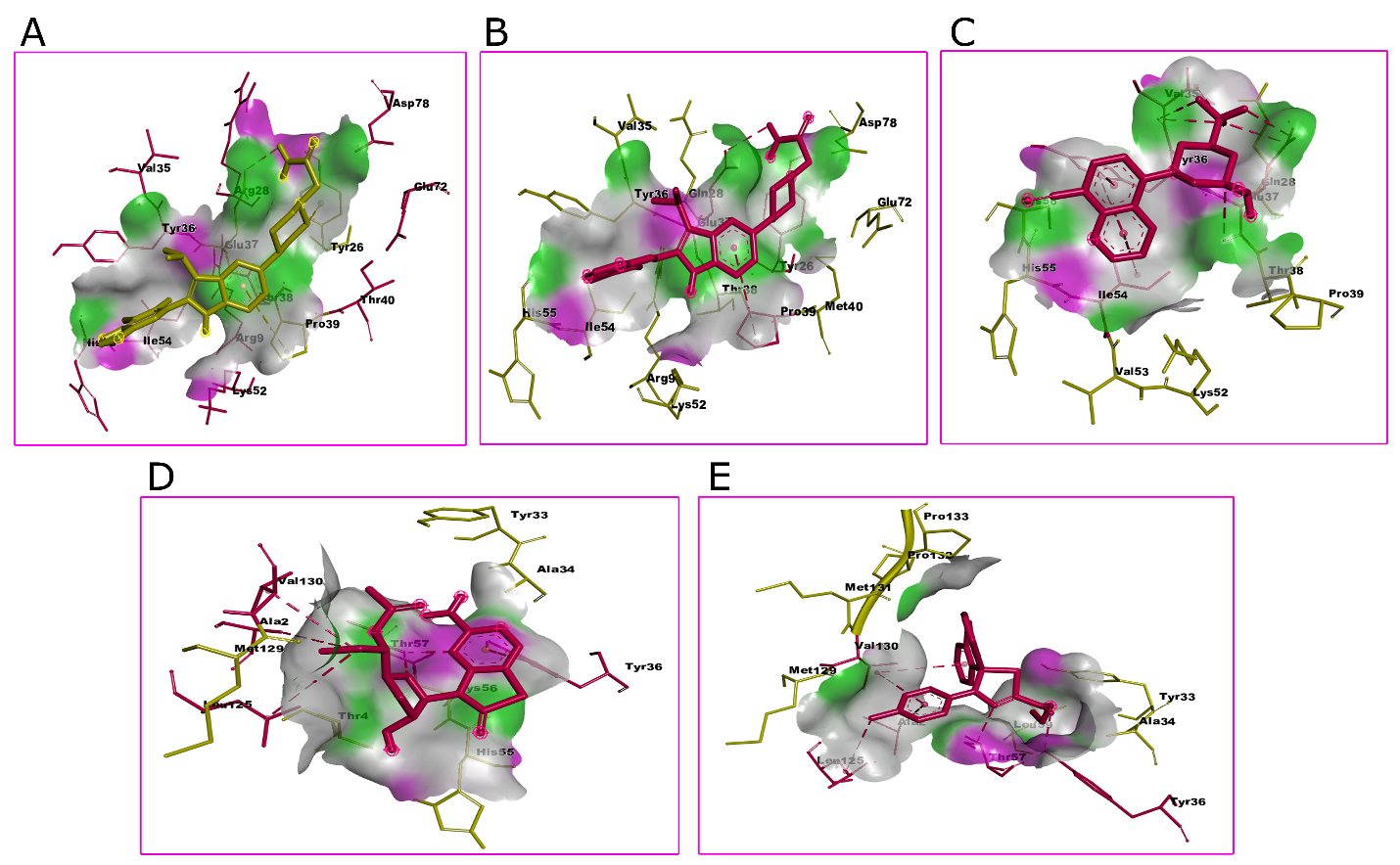


**Figure S4:** The 2D surface around drug ligand. (A) Wild, (B) R28Q, (C) T40M, (D) P59L, (E) R200C.
